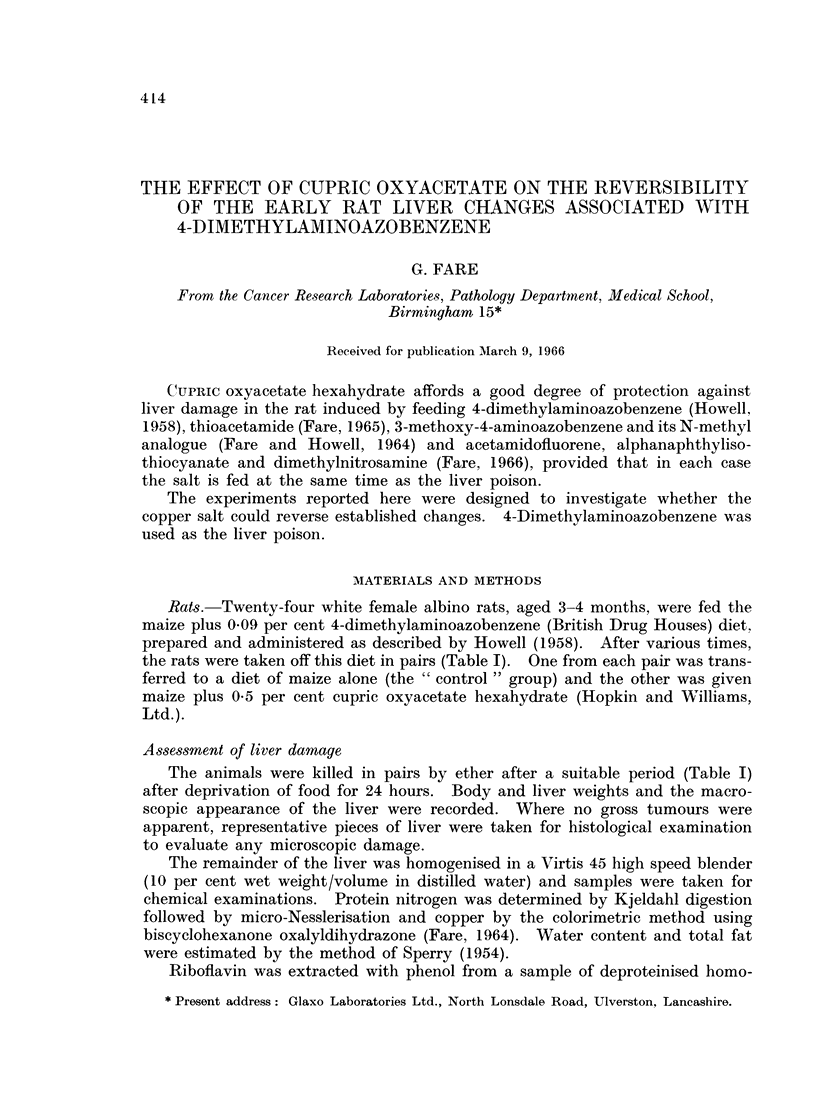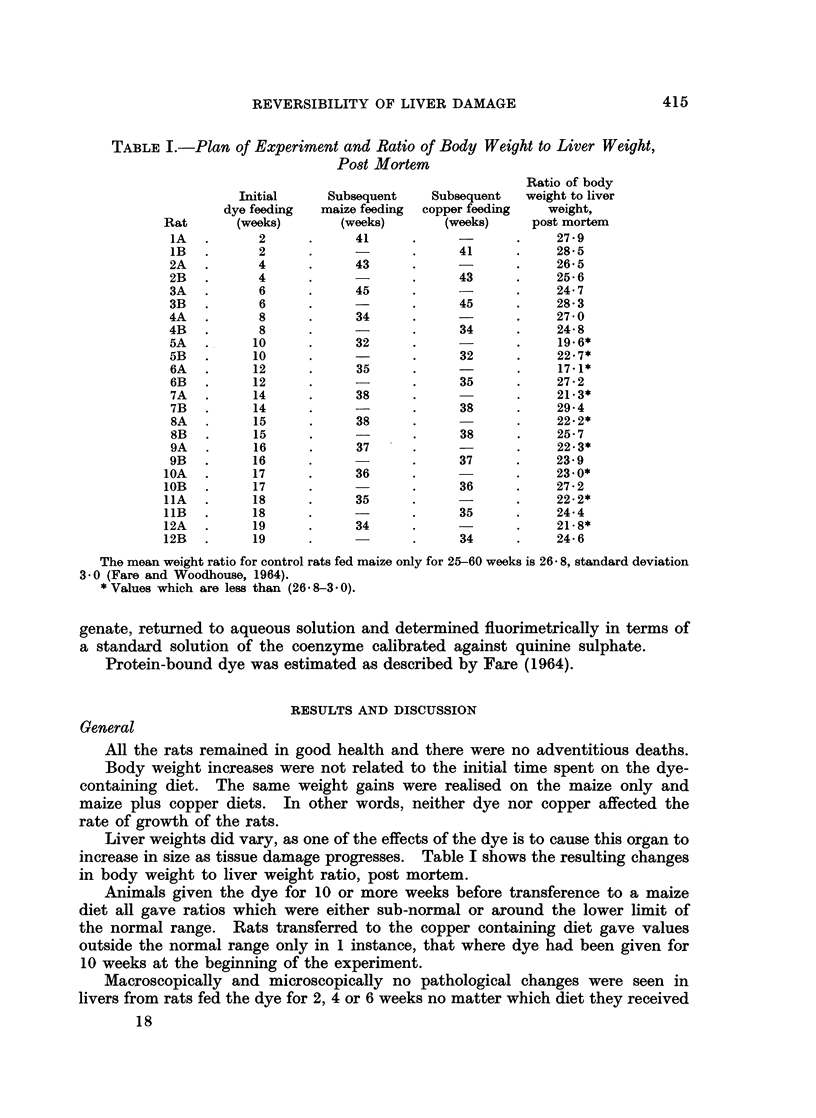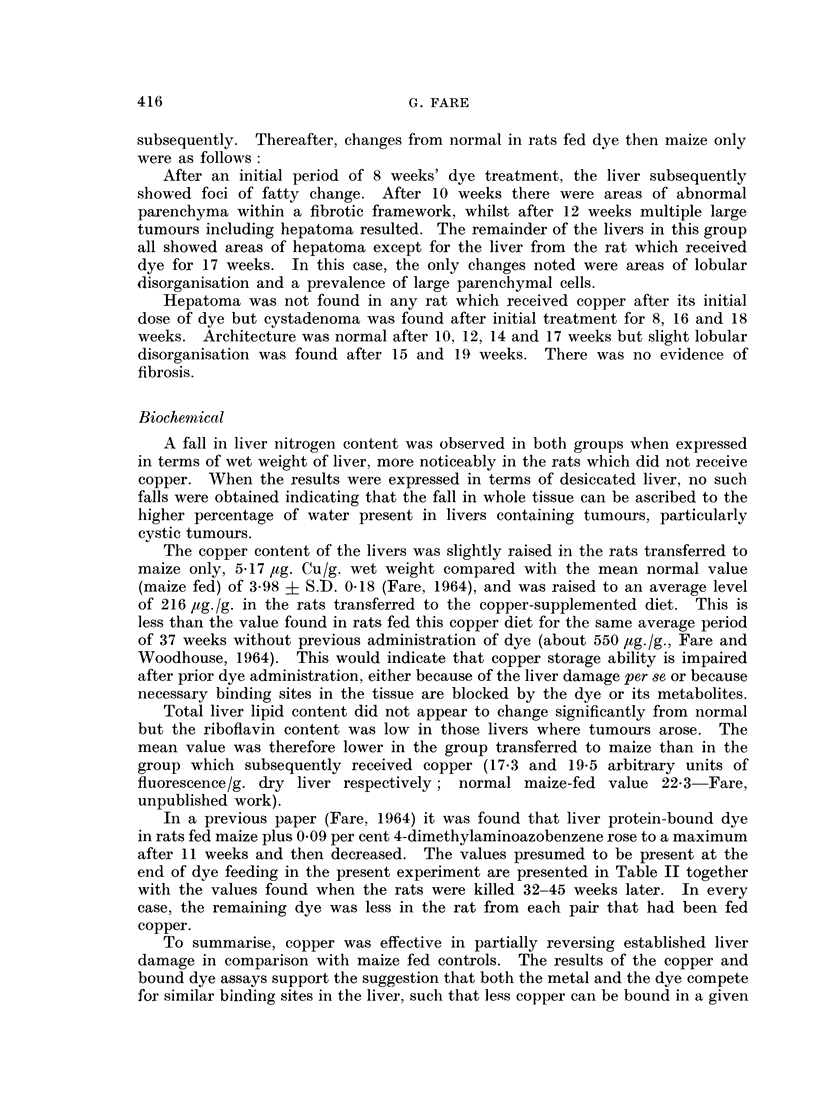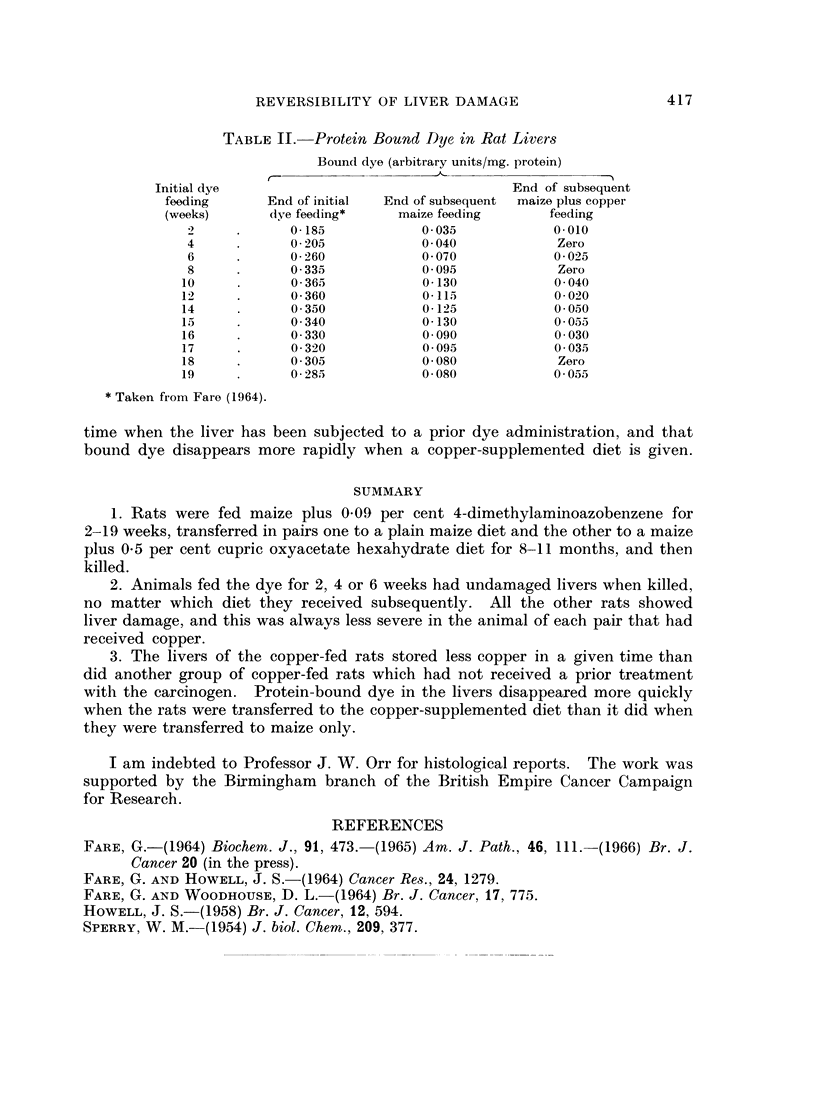# The effect of cupric oxyacetate on the reversibility of the early rat liver changes associated with 4-dimethylaminoazobenzene.

**DOI:** 10.1038/bjc.1966.49

**Published:** 1966-06

**Authors:** G. Fare


### THE EFFECT OF CUPRIC OXYACETATE ON THE REVERSIBILITY OF THE EARLY RAT LIVER CHANGES ASSOCIATED WITH 4-DIMETHYLAMINOAZOBENZENE

G. FARE

*From the Cancer Research Laboratories, Pathology Department, Medical School, Birmingham* 15*

Received for publication March 9, 1966

CUPRIC oxyacetate hexahydrate affords a good degree of protection against liver damage in the rat induced by feeding 4-dimethylaminoazobenzene (Howell, 1958), thioacetamide (Fare, 1965), 3-methoxy-4-aminoazobenzene and its N-methyl analogue (Fare and Howell, 1964) and acetamidofluorene, alphanaphthyliso-thiocyanate and dimethylnitrosamine (Fare, 1966), provided that in each case the salt is fed at the same time as the liver poison.

The experiments reported here were designed to investigate whether the copper salt could reverse established changes. 4-Dimethylaminoazobenzene was used as the liver poison.

### MATERIALS AND METHODS

*Rats.*—Twenty-four white female albino rats, aged 3–4 months, were fed the maize plus 0·09 per cent 4-dimethylaminoazobenzene (British Drug Houses) diet, prepared and administered as described by Howell (1958). After various times, the rats were taken off this diet in pairs (Table I). One from each pair was transferred to a diet of maize alone (the " control " group) and the other was given maize plus 0·5 per cent cupric oxyacetate hexahydrate (Hopkin and Williams, Ltd.).

### *Assessment of liver damage*

The animals were killed in pairs by ether after a suitable period (Table I) after deprivation of food for 24 hours. Body and liver weights and the macroscopic appearance of the liver were recorded. Where no gross tumours were apparent, representative pieces of liver were taken for histological examination to evaluate any microscopic damage.

The remainder of the liver was homogenised in a Virtis 45 high speed blender (10 per cent wet weight/volume in distilled water) and samples were taken for chemical examinations. Protein nitrogen was determined by Kjeldahl digestion followed by micro-Nesslerisation and copper by the colorimetric method using biscyclohexanone oxalyldihydrazone (Fare, 1964). Water content and total fat were estimated by the method of Sperry (1954).

Riboflavin was extracted with phenol from a sample of deproteinised homo-

* Present address : Glaxo Laboratories Ltd., North Lonsdale Road, Ulverston, Lancashire.

TABLE I.—*Plan of Experiment and Ratio of Body Weight to Liver Weight, Post Mortem*

| Rat | Initial dye feeding (weeks) | Subsequent maize feeding (weeks) | Subsequent copper feeding (weeks) | Ratio of body weight to liver weight, post mortem |
|---|---|---|---|---|
| 1A | 2 | 41 | — | 27·9 |
| 1B | 2 | — | 41 | 28·5 |
| 2A | 4 | 43 | — | 26·5 |
| 2B | 4 | — | 43 | 25·6 |
| 3A | 6 | 45 | — | 24·7 |
| 3B | 6 | — | 45 | 28·3 |
| 4A | 8 | 34 | — | 27·0 |
| 4B | 8 | — | 34 | 24·8 |
| 5A | 10 | 32 | — | 19·6* |
| 5B | 10 | — | 32 | 22·7* |
| 6A | 12 | 35 | — | 17·1* |
| 6B | 12 | — | 35 | 27·2 |
| 7A | 14 | 38 | — | 21·3* |
| 7B | 14 | — | 38 | 29·4 |
| 8A | 15 | 38 | — | 22·2* |
| 8B | 15 | — | 38 | 25·7 |
| 9A | 16 | 37 | — | 22·3* |
| 9B | 16 | — | 37 | 23·9 |
| 10A | 17 | 36 | — | 23·0* |
| 10B | 17 | — | 36 | 27·2 |
| 11A | 18 | 35 | — | 22·2* |
| 11B | 18 | — | 35 | 24·4 |
| 12A | 19 | 34 | — | 21·8* |
| 12B | 19 | — | 34 | 24·6 |

The mean weight ratio for control rats fed maize only for 25–60 weeks is 26·8, standard deviation 3·0 (Fare and Woodhouse, 1964).
* Values which are less than (26·8–3·0).

genate, returned to aqueous solution and determined fluorimetrically in terms of a standard solution of the coenzyme calibrated against quinine sulphate.

Protein-bound dye was estimated as described by Fare (1964).

### RESULTS AND DISCUSSION

### *General*

All the rats remained in good health and there were no adventitious deaths.

Body weight increases were not related to the initial time spent on the dye-containing diet. The same weight gains were realised on the maize only and maize plus copper diets. In other words, neither dye nor copper affected the rate of growth of the rats.

Liver weights did vary, as one of the effects of the dye is to cause this organ to increase in size as tissue damage progresses. Table I shows the resulting changes in body weight to liver weight ratio, post mortem.

Animals given the dye for 10 or more weeks before transference to a maize diet all gave ratios which were either sub-normal or around the lower limit of the normal range. Rats transferred to the copper containing diet gave values outside the normal range only in 1 instance, that where dye had been given for 10 weeks at the beginning of the experiment.

Macroscopically and microscopically no pathological changes were seen in livers from rats fed the dye for 2, 4 or 6 weeks no matter which diet they received subsequently. Thereafter, changes from normal in rats fed dye then maize only were as follows :

After an initial period of 8 weeks' dye treatment, the liver subsequently showed foci of fatty change. After 10 weeks there were areas of abnormal parenchyma within a fibrotic framework, whilst after 12 weeks multiple large tumours including hepatoma resulted. The remainder of the livers in this group all showed areas of hepatoma except for the liver from the rat which received dye for 17 weeks. In this case, the only changes noted were areas of lobular disorganisation and a prevalence of large parenchymal cells.

Hepatoma was not found in any rat which received copper after its initial dose of dye but cystadenoma was found after initial treatment for 8, 16 and 18 weeks. Architecture was normal after 10, 12, 14 and 17 weeks but slight lobular disorganisation was found after 15 and 19 weeks. There was no evidence of fibrosis.

*Biochemical*

A fall in liver nitrogen content was observed in both groups when expressed in terms of wet weight of liver, more noticeably in the rats which did not receive copper. When the results were expressed in terms of desiccated liver, no such falls were obtained indicating that the fall in whole tissue can be ascribed to the higher percentage of water present in livers containing tumours, particularly cystic tumours.

The copper content of the livers was slightly raised in the rats transferred to maize only, 5·17 $\mu$g. Cu/g. wet weight compared with the mean normal value (maize fed) of 3·98 $\pm$ S.D. 0·18 (Fare, 1964), and was raised to an average level of 216 $\mu$g./g. in the rats transferred to the copper-supplemented diet. This is less than the value found in rats fed this copper diet for the same average period of 37 weeks without previous administration of dye (about 550 $\mu$g./g., Fare and Woodhouse, 1964). This would indicate that copper storage ability is impaired after prior dye administration, either because of the liver damage *per se* or because necessary binding sites in the tissue are blocked by the dye or its metabolites.

Total liver lipid content did not appear to change significantly from normal but the riboflavin content was low in those livers where tumours arose. The mean value was therefore lower in the group transferred to maize than in the group which subsequently received copper (17·3 and 19·5 arbitrary units of fluorescence/g. dry liver respectively ; normal maize-fed value 22·3—Fare, unpublished work).

In a previous paper (Fare, 1964) it was found that liver protein-bound dye in rats fed maize plus 0·09 per cent 4-dimethylaminoazobenzene rose to a maximum after 11 weeks and then decreased. The values presumed to be present at the end of dye feeding in the present experiment are presented in Table II together with the values found when the rats were killed 32–45 weeks later. In every case, the remaining dye was less in the rat from each pair that had been fed copper.

To summarise, copper was effective in partially reversing established liver damage in comparison with maize fed controls. The results of the copper and bound dye assays support the suggestion that both the metal and the dye compete for similar binding sites in the liver, such that less copper can be bound in a given

TABLE II.—*Protein Bound Dye in Rat Livers*

| Initial dye feeding (weeks) | Bound dye (arbitrary units/mg. protein) | | |
|---|---|---|---|
| | End of initial dye feeding* | End of subsequent maize feeding | End of subsequent maize plus copper feeding |
| 2 | 0·185 | 0·035 | 0·010 |
| 4 | 0·205 | 0·040 | Zero |
| 6 | 0·260 | 0·070 | 0·025 |
| 8 | 0·335 | 0·095 | Zero |
| 10 | 0·365 | 0·130 | 0·040 |
| 12 | 0·360 | 0·115 | 0·020 |
| 14 | 0·350 | 0·125 | 0·050 |
| 15 | 0·340 | 0·130 | 0·055 |
| 16 | 0·330 | 0·090 | 0·030 |
| 17 | 0·320 | 0·095 | 0·035 |
| 18 | 0·305 | 0·080 | Zero |
| 19 | 0·285 | 0·080 | 0·055 |

* Taken from Fare (1964).

time when the liver has been subjected to a prior dye administration, and that bound dye disappears more rapidly when a copper-supplemented diet is given.

### SUMMARY

1. Rats were fed maize plus 0·09 per cent 4-dimethylaminoazobenzene for 2–19 weeks, transferred in pairs one to a plain maize diet and the other to a maize plus 0·5 per cent cupric oxyacetate hexahydrate diet for 8–11 months, and then killed.

2. Animals fed the dye for 2, 4 or 6 weeks had undamaged livers when killed, no matter which diet they received subsequently. All the other rats showed liver damage, and this was always less severe in the animal of each pair that had received copper.

3. The livers of the copper-fed rats stored less copper in a given time than did another group of copper-fed rats which had not received a prior treatment with the carcinogen. Protein-bound dye in the livers disappeared more quickly when the rats were transferred to the copper-supplemented diet than it did when they were transferred to maize only.

I am indebted to Professor J. W. Orr for histological reports. The work was supported by the Birmingham branch of the British Empire Cancer Campaign for Research.

### REFERENCES

FARE, G.—(1964) *Biochem. J.*, **91**, 473.—(1965) *Am. J. Path.*, **46**, 111.—(1966) *Br. J. Cancer* **20** (in the press).
FARE, G. AND HOWELL, J. S.—(1964) *Cancer Res.*, **24**, 1279.
FARE, G. AND WOODHOUSE, D. L.—(1964) *Br. J. Cancer*, **17**, 775.
HOWELL, J. S.—(1958) *Br. J. Cancer*, **12**, 594.
SPERRY, W. M.—(1954) *J. biol. Chem.*, **209**, 377.